# Supplementary material for: Influence of the COVID-19 pandemic on breastfeeding support for healthy mothers and the association between compliance with WHO recommendations for breastfeeding support and exclusive breastfeeding in Japan
Source: PeerJ. 2022 May 19;10:e13347. doi: 10.7717/peerj.13347 (PMC9124456; doi:10.7717/peerj.13347)
Supplement: Supplemental Information 2 — The left column shows the summary of each step. The right column explains how each step was measured or why the step was not measured. [file peerj-10-13347-s002.rtf]

Definitions and measurement of the ten steps to successful breastfeeding

Stepa	Measurement	
Step 1a. Comply fully with the International Code of Marketing of Breast-milk Substitutes and relevant World Health Assembly resolutions. 1b. Have a written infant feeding policy that is routinely communicated to staff and parents.1c. Establish ongoing monitoring and data-management systems.	Not measured. It relates to the hospital policy, staff training, and monitoring system and thus is not considered measurable by maternal reports.	
Step 2. Ensure that staff have sufficient knowledge, competence and skills to support breastfeeding.	Not measured. It was considered not measurable by maternal reports.	
Step 3. Discuss the importance and management of breastfeeding with pregnant women and their families.	Mothers were asked, “Did you learn the importance and management of breastfeeding during the pregnancy?” The answers “taught enough,” “taught to some extent,” and “knew enough so did not seek help” were considered receipt of Step 3; “not taught” and “never taught much” were considered nonreceipt of Step 3.	
Step 4. Facilitate immediate and uninterrupted skin-to-skin contact and support mothers to initiate breastfeeding as soon as possible after birth.	Mothers were asked, “Did you initiate breastfeeding within about 30 minutes after birth?” The answer “yes” was considered receipt of, and the answer “no” was considered nonreceipt of, Step 4.	
Step 5. Support mothers to initiate and maintain breastfeeding and manage common difficulties.	Mothers were asked, “After giving birth, did your midwife or doctor teach you how to breastfeed in a way you wanted to?” The answers “taught enough,” “taught to some extent,” and “knew enough so did not seek help” were considered receipt of Step 5; the answers “not taught” and “never taught much” were considered nonreceipt of Step 5.	
Step 6. Do not provide breastfed newborns any food or fluids other than breast milk, unless medically indicated.	Not measured because mothers might be unable to determine if supplementation was indicated.	
Step 7. Enable mothers and their infants to remain together and to practice rooming-in 24 hours a day.	Mothers were asked, “Did you stay with your baby in the same room during the day and night since soon after the birth?” The answer “yes” was considered receipt of, and the answer “no” was considered nonreceipt of, Step 7.	
Step 8. Support mothers to recognize and respond to their infants' cues for feeding.	Mothers were asked, “At the facility, did you breastfeed your baby whenever he or she wanted to?” The answer “yes” was considered receipt of, and the answer “no” was considered nonreceipt of, Step 8.	
Step 9. Counsel mothers on the use and risks of feeding bottles, teats and pacifiers.	Not measured because variability in responses was expected to be minimal. In Japan, there is no legal regulation of the marketing of breast milk substitutes and its accessory products at hospitals or anywhere else. Bottles are common in Japanese hospitals.	
Step 10. Coordinate discharge so that parents and their infants have timely access to ongoing support and care.	Not measured because variability in responses was expected to be minimal. All postpartum mothers in Japan are expected to receive support through home visits and regular infant health checkups by public health center staff.	
a Summary of the steps described on the WHO website at the URL below. Authors accessed on 16 October 2021. 
https://www.who.int/teams/nutrition-and-food-safety/food-and-nutrition-actions-in-health-systems/ten-steps-to-successful-breastfeeding
