# Supplementary material for: Influence of the COVID-19 pandemic on breastfeeding support for healthy mothers and the association between compliance with WHO recommendations for breastfeeding support and exclusive breastfeeding in Japan
Source: PeerJ. 2022 May 19;10:e13347. doi: 10.7717/peerj.13347 (PMC9124456; doi:10.7717/peerj.13347)
Supplement: Supplemental Information 3 [file peerj-10-13347-s003.docx]

Questionnaire with English translation

|  | Question | Answer choices | English translation of the question | English translation of the answer choices |
| --- | --- | --- | --- | --- |
| JAC20P_Q13 | 現在、配偶者は、いますか。 配偶者には、事実上夫婦として生活しているが、婚姻届を提出していない場合も含みます。 | 1 配偶者あり 2 未婚 3 死別（2020年3月以前に死別した） 4 死別（2020年4月以降に死別した） 5 離婚（2020年3月以前に離婚した） 6 離婚（2020年4月以降に離婚した） | Do you currently have a spouse? Spouse includes de fact partner. | 1 Spouse present 2 Unmarried 3 Bereaved (bereaved before March 2020) 4 Bereaved (bereaved after April 2020) 5 Divorced (divorced before March 2020) 6 Divorced (divorced after April 2020)" |
| JAC20P_Q20 | 現在のあなたの仕事の状況についてお答えください。 | 1 主に職場で仕事をしている 2 主に自宅で仕事をしている 3 産休もしくは育休中 4 休暇もしくは自宅待機中 5 妊娠前まで仕事していたが、妊娠・出産をきっかけに仕事をやめた 6 妊娠前から、仕事をしていない | Please choose your current working status. | 1 I work mainly outside home 2 I work mainly from home 3 On maternity or paternity leave 4 On leave or staying home 5 Was working before pregnancy, but quitted after pregnancy or childbirth 6 Not working since before pregnancy |
| JAC20P_Q213 | 妊婦健診の回数を減らした | 1 はい 2 いいえ | I reduced the frequency of prenatal checkups. | 1 Yes 2 No |
| JAC20P_Q214 | 妊娠期間中、母親学級や両親学級を受講できなかった、あるいは減らした | 1 はい 2 いいえ | I was unable to attend/I reduced the number of maternity/parent education classes during pregnancy. | 1 Yes 2 No |
| JAC20P_Q221 | 出産時、夫の立ち合いはしなかった | 1 はい 2 いいえ | My husband was not present during childbirth. | 1 Yes 2 No |
| JAC20P_Q222 | （出産後の）入院中、あなたや赤ちゃんへの家族の面会が制限された | 1 はい 2 いいえ | During the hospital stay after delivery, my family was not allowed to visit me and my baby. | 1 Yes 2 No |
| JAC20P_Q223 | （出産後の）入院中、医師、助産師、看護師から育児や授乳について教えてもらう機会が少なかった | 1 はい 2 いいえ | During the hospital stay after delivery, I did not have enough opportunities to learn about childcare and breastfeeding from doctors, midwives, or nurses. | 1 Yes 2 No |
| JAC20P_Q224 | 出産後、親や他の家族に手伝いに来てもらうことができなかった | 1 はい 2 いいえ | I was not able to ask my parents or other family members to come and help me after discharge. | 1 Yes 2 No |
| JAC20P_Q225 | 出産後、親・義親宅に滞在する予定だったが、取りやめた | 1 はい 2 いいえ | Planned to stay with parents or parents-in-law after discharge, but decided not to. | 1 Yes 2 No |
| JAC20P_Q226 | 出産後、あなた自身の健康について医師や助産師に相談したいことがあったが、 できなかった（または、受診を控えた） | 1 はい 2 いいえ | I wanted to consult a doctor or midwife about my health after discharge, but was unable to do so (or refrained from seeing a doctor)." | 1 Yes 2 No |
| JAC20P_Q227 | 出産後、保健師や助産師等による家庭訪問を希望したにもかかわらず、受けられなかった | 1 はい 2 いいえ | I requested a home visit by a public health nurse or midwife after discharge but did not receive the service. | 1 Yes 2 No |
| JAC20P_Q228 | 出産後、保健師や助産師等による家庭訪問を受けることを自発的に控えた | 1 はい 2 いいえ | I refrained from receiving a home visit by a public health nurse or midwife after discharge. | 1 Yes 2 No |
| JAC20P_Q2213 | 授乳や育児の悩みについて友人・知人に相談したかったが、できなかった | 1 はい 2 いいえ | I wanted to talk to a friend or acquaintance about my concerns with breastfeeding or childcare, but was unable to do so | 1 Yes 2 No |
| JAC20P_Q241 | 出生時の体重が2,500g未満 | 1 はい 2 いいえ | Birth weight was less than 2,500g | 1 Yes 2 No |
| JAC20P_Q242 | 妊娠37週未満で出生 | 1 はい 2 いいえ | Born before 37 weeks of gestation | 1 Yes 2 No |
| JAC20P_Q243 | 赤ちゃんが、新生児集中治療室（NICU）に入院した | 1 はい 2 いいえ | My baby was admitted to the Neonatal Intensive Care Unit (NICU) | 1 Yes 2 No |
| JAC20P_Q244 | 出産後、あなた自身の健康状態が悪かったため、赤ちゃんに会えない日があった | 1 はい 2 いいえ | There were days after the birth when I could not see my baby because of my poor health status | 1 Yes 2 No |
| JAC20P_Q25 | 母乳で育てることをどう思っていますか。  ※すでに出産された方は、妊娠中の時にどう思っていたかお答えください。 | 1 ぜひ母乳で育てたいと思っている（思っていた） 2 母乳がでれば母乳で育てたいと思っている（思っていた） 3 ミルクで育てたいと思っている（思っていた） 4 母乳とミルクの両方で育てたいと思っている（思っていた） 5 特に考えていない（考えなかった） | What is your plan about infant feeding?  *If you have already given birth, please answer how you thought about it when you were pregnant. | 1 I definitely like to breastfeed my child (thought so). 2 I want to breastfeed my baby if I can (thought so). 3 I want to raise my child with milk (thought so) 4 I want to raise my child with both breast milk and formula (thought so) 5 I have no thought about it in particular (did not have a particular plan). |
| JAC20P_Q261 | お子さんを母乳で育てることについて、医療機関等で、妊娠中に利点や具体的なやり方を教わりましたか | 1 十分教わった 2 ある程度教わった 3 あまり教わらなかった 4 まったく教わらなかった 5 必要がなかったので、教わらなかった | Were you taught about the benefits of and necessary skills to breastfeed your child at a medical institution during your pregnancy? | 1 I was taught enough 2 I was taught to some extent 3 I was not taught much 4 I was not taught at all 5 I did not seek help because I did not need it. |
| JAC20P_Q262 | 出産後、授乳について困ったときに医療機関等で具体的に対処方法を教わりましたか | 1 十分教わった 2 ある程度教わった 3 あまり教わらなかった 4 まったく教わらなかった 5 必要がなかったので、教わらなかった | After giving birth, when you had trouble with breastfeeding, did the medical staff teach you how to deal with it? | 1 I was taught enough 2 I was taught to some extent 3 I was not taught much 4 I was not taught at all 5 I did not seek help because I did not need it. |
| JAC20P_Q263 | 出産後、あなた自身が希望している授乳方法を実践できるように、助産師や医師からやり方を教わることができましたか | 1 十分教わった 2 ある程度教わった 3 あまり教わらなかった 4 まったく教わらなかった 5 必要がなかったので、教わらなかった | After giving birth, did you learn how to breastfeed your baby the way you wanted to? | 1 I was taught enough 2 I was taught to some extent 3 I was not taught much 4 I was not taught at all 5 I did not seek help because I did not need it. |
| JAC20P_Q271 | 出産した医療施設で、 出産後30分以内に母乳を飲ませましたか | 1 はい 2 いいえ | At the medical facility where you gave birth, did  you breastfeed your baby within 30 minutes of delivery? | 1 Yes 2 No |
| JAC20P_Q272 | 出産した医療施設では、 出産直後から母子同室でしたか | 1 はい 2 いいえ | At the medical facility where you gave birth, did you stay in the same room with your baby since immediately after delivery?" | 1 Yes 2 No |
| JAC20P_Q273 | 出産した医療施設では、 赤ちゃんが欲しがる時はいつでも母乳を飲ませましたか | 1 はい 2 いいえ | At the medical facility where you gave birth, did you breastfeed your baby whenever he or she wanted to? | 1 Yes 2 No |
| JAC20P_Q279 | 粉ミルクや液体ミルクの無料配布キャンペーンを目にしたり、 無料配布を受け取ったりしたことがありますか | 1 はい 2 いいえ | Have you ever seen a campaign to distribute free formula or liquid milk or received a free sample of formula or liquid milk? | 1 Yes 2 No |
| JAC20P_Q281 | 昨日から今日までの24時間に、赤ちゃんに次のものを与えましたか。1. 母乳 | 1 はい 2 いいえ | In the 24 hours from yesterday to today, did you give your baby the following: 1. breast milk | 1 Yes 2 No |
| JAC20P_Q282 | 昨日から今日までの24時間に、赤ちゃんに次のものを与えましたか。2．ミルク（乳児用の粉ミルクや液体ミルクなど） | 1 はい 2 いいえ | In the past 24 hours, did you give your baby: 1. breast milk 2. formula (such as infant formula or liquid milk for babies) | 1 Yes 2 No |
| JAC20P_Q283 | 昨日から今日までの24時間に、赤ちゃんに次のものを与えましたか。３．母乳とミルク以外の飲み物（お茶、白湯、果汁、イオン飲料など） | 1 はい 2 いいえ | In the 24 hours between yesterday and today, did you give your baby any of the following: 3. drinks other than breast milk and formula (tea, white water, fruit juice, ionized drink, etc.) | 1 Yes 2 No |
| JAC20P_Q284 | 昨日から今日までの24時間に、赤ちゃんに次のものを与えましたか。4．離乳食 （おかゆ、野菜、豆腐、魚、肉、果物など） | 1 はい 2 いいえ | In the 24 hours between yesterday and today, did you give your baby any of the following: 4. baby food (porridge, vegetables, tofu, fish, meat, fruit, etc.) | 1 Yes 2 No |
| JAC20P_Q29S1 | Edinburgh postnatal depression scale, item 1 |  | Edinburgh postnatal depression scale, item 1 |  |
| JAC20P_Q29S2 | Edinburgh postnatal depression scale, item 2 |  | Edinburgh postnatal depression scale, item 2 |  |
| JAC20P_Q29S3 | Edinburgh postnatal depression scale, item 3 |  | Edinburgh postnatal depression scale, item 3 |  |
| JAC20P_Q29S4 | Edinburgh postnatal depression scale, item 4 |  | Edinburgh postnatal depression scale, item 4 |  |
| JAC20P_Q29S5 | Edinburgh postnatal depression scale, item 5 |  | Edinburgh postnatal depression scale, item 5 |  |
| JAC20P_Q29S6 | Edinburgh postnatal depression scale, item 6 |  | Edinburgh postnatal depression scale, item 6 |  |
| JAC20P_Q29S7 | Edinburgh postnatal depression scale, item 7 |  | Edinburgh postnatal depression scale, item 7 |  |
| JAC20P_Q29S8 | Edinburgh postnatal depression scale, item 8 |  | Edinburgh postnatal depression scale, item 8 |  |
| JAC20P_Q29S9 | Edinburgh postnatal depression scale, item 9 |  | Edinburgh postnatal depression scale, item 9 |  |
| JAC20P_Q29S10 | Edinburgh postnatal depression scale, item 10 |  | Edinburgh postnatal depression scale, item 10 |  |
| JAC20P_Q306 | あなたのパートナー（夫や事実婚の相手）は、 あなたが困っているときに助けてくれますか | 1 はい、いつもそうです 2 はい、ときどきそうです 3 いいえ、あまりそうではありません 4 いいえ、まったくそうではありません | Does your partner (husband or de facto partner) support you when you are in trouble? | 1 Yes, always 2 Yes, sometimes 3 No, not often 4 No, not at all |
| JAC20P_Q307 | あなたのパートナーは、あなたの気持ち （うれしさ、楽しさ、悲しさ、怒りなど）に応えてくれますか | 1 はい、いつもそうです 2 はい、ときどきそうです 3 いいえ、あまりそうではありません 4 いいえ、まったくそうではありません | Does your partner respond to your feelings (happiness, joy, sadness, anger, etc.)? | 1 Yes, always 2 Yes, sometimes 3 No, not often 4 No, not at all |
| JAC20P_Q308 | あなたのパートナーは、家事や育児など家庭での役割を果たしてくれますか | 1 はい、いつもそうです 2 はい、ときどきそうです 3 いいえ、あまりそうではありません 4 いいえ、まったくそうではありません | Does your partner fulfill his role in the household, such as housework and childcare? | 1 Yes, always 2 Yes, sometimes 3 No, not often 4 No, not at all |
